# Extra-hepatic portal vein obstruction and type 1 diabetes in a child: a co-incidence or causal association?

**DOI:** 10.1186/1687-9856-2013-S1-P37

**Published:** 2013-10-03

**Authors:** Rakesh Kumar, Devi Dayal

**Affiliations:** 1 Advanced Pediatric Centre, Post Graduate Institute of Medical Education and Research, Sector 12, Chandigarh, India – 160012

## Introduction

We report an eight year old boy who had extra hepatic portal vein obstruction (EHPVO) and developed type1 diabetes (type1 DM).

## Case report

An eight year old boy presented with polyuria and polydipsia for 1 month. On examination, liver was not palpable but spleen was enlarged (6cm). Blood sugar was 460 mg/dl and type1 DM was confirmed. Ultrasonography showed portal vein cavernoma with normal liver and pancreas. Endoscopy revealed grade 2 esophageal varices. Thus diagnosis of EHPVO with portal hypertension was also confirmed. Other investigations including liver functions, hemogram, renal functions, serum amylase, lipid profile, thyroid function, Protein C, S and factor V Laiden estimation were normal. There was no history of abdominal infections like peritonitis, pancreatitis and umbilical sepsis or umbilical artery catheterization in neonatal period. C-peptide level was low (0.111 ng/ml) with weakly positive Islet cell antibodies, positive GAD 65 antibodies (> 2000 IU/ml). Tissue peroxidase and Tissue transglutaminase antibodies were negative. Patient is asymptomatic after endoscopic band ligation of esophageal varices and split-mix insulin regimen.

## Discussion

In the index case sequence of events seems to be portal vein thrombosis at least few years before, leading then to cavernoma formation and narrowing of portal vein leading to development of portal hypertension over the years which remained asymptomatic till child developed diabetes. Similar sequence was seen in two previously reported patients of EHPVO who went on to develop type 1 diabetes on follow up of few years [1]. Only common condition which can cause both EHPVO and type1 diabetes is pancreatitis. However, there is no proof of pancreatitis in the index case. Authors in above report have also suggested that any disease in portal vein may incite inflammatory response in the pancreas [1]. In above mentioned report of three cases, authors have reported 3 cases of Type1 DM in a cohort of 100 cases of EHPVO and in our cohort of around 500 type1 DM patients this is the first case identified to have EHPVO.To conclude, this is only the fourth case reported in literature, to have EHPVO who developed type1 DM. In the index case it looks more of a co-incidence rather than a causal association between the two entities.
